# Supplementary material for: Turnip mosaic virus co-opts the vacuolar sorting receptor VSR4 to promote viral genome replication in plants by targeting viral replication vesicles to the endosome
Source: PLoS Pathog. 2022 Jan 24;18(1):e1010257. doi: 10.1371/journal.ppat.1010257 (PMC8812904; doi:10.1371/journal.ppat.1010257)
Supplement: S1 Table — (DOCX) [file ppat.1010257.s001.docx]

**S1 Table.** Primers used in this study.

| **Primer names** | **Sequences (5’ to 3’)** | **Notes** |
| --- | --- | --- |
| pPR3-N-AtVSR4-F | GAGTGGCCATTACGGCCatgaagcagcttctatgttatct | Primers are designed for cloning into vector pPR3-N,  and designed based on the reference sequence NM_127036.5. |
| pPR3-N- AtVSR4-R | GAGAGGCCGAGGCGGCCGTTAggcacgttcatcattcgtgtg |  |
| BP-AtVSR4-F | GGGGACAAGTTTGTACAAAAAAGCAGGCTTCatgaagcagcttctatgttatct | Primers are designed for cloning into the entry vector pDONR221.  GenBank Accession no.: NM_127036.5 |
| BP-AtVSR4-R | GGGGACCACTTTGTACAAGAAAGCTGGGTCggcacgttcatcattcgtgtg |  |
| BP-AtVSR4_STOP_-R | GGGGACCACTTTGTACAAGAAAGCTGGGTCttaggcacgttcatcattcgtgtg |  |
| BP-AtVSR4-LD-R | GGGGACCACTTTGTACAAGAAAGCTGGGTCtgatttcacttgtgaacccgtc | For generation of AtDRP1A truncated mutants for each domain. |
| BP-AtVSR4-TMD-F | GGGGACAAGTTTGTACAAAAAAGCAGGCTTCatggcgtgggcggccgtttggcttataatg |  |
| BP-AtVSR4-TMD-R | GGGGACCACTTTGTACAAGAAAGCTGGGTCgtaaacgaggtatgcaccag |  |
| BP-AtVSR4-CT-F | GGGGACAAGTTTGTACAAAAAAGCAGGCTTCatgaaatatagattgaggcaatac |  |
| atvsr4-LP (SALK_094467C) | GTGAAGGATTTTAAGGGAGCG | For PCR genotyping. |
| atvsr4-LP (SALK_094467C) | TTTTCCACGAAACGTATCCTG |  |
| atvsr1-LP (SALK_040506C) | ATTTGATGAATGTGGTGGTGG |  |
| atvsr1-LP (SALK_040506C) | ACCTCCACGATCGATAAGGAC |  |
| atvsr2-LP (SALK_150241) | TATGTTCGGAGACGACGAAAC |  |
| atvsr2-LP (SALK_150241) | GAGTGTGAGGAGAAAACAGCG |  |
| atvsr5-LP (SALK_044991C) | AAACCAAGGGAGGTATTGTGC |  |
| atvsr5-LP (SALK_044991C) | AAAACATACGTTTGCAGGAGG |  |
| atvsr7-LP (SALK_202877) | AGTGCCTTGAAAACAATGGTG |  |
| atvsr7-LP (SALK_202877) | CTGATGGAACTTCACGAGCTC |  |
| LBb1.3 | ATTTTGCCGATTTCGGAAC |  |
| qPCR-TuMV cp-F | TGGCTGATTACGAACTGACG | Primers are designed based on the submitted NCBI sequence for the TuMV isolate (EF028235.1). |
| qPCR-TuMV cp-R | CTGCCTAAATGTGGGTTTGG |  |
| qPCR-TuMV nib-F | GTGGAAAAGTTTGCGTGGAT |  |
| qPCR-TuMV nib-R | CATCAGCATCGCAATACACC |  |
| qPCR-AtVSR4-F | AAGCGGGCCAGGGAGATGTA | Primers are designed based on the reference sequences (NM_127036.5 for AtVSR4 deposited in GenBank). |
| qPCR-AtVSR4-R | TCCGGGCACTGACATGCTTT |  |
| qPCR-AtActin II-F | CACCACAACAGCAGAGCGGGA | Primers are designed based on the reference sequences (NM_112764.4 for AtACT II deposited in GenBank). |
| qPCR-AtActin II-R | TCCCACAAACGAGGGCTGGA |  |
| qPCR-Nbactin-F | GGGATGTGAAGGAGAAGTTGGC | Primers are designed based on the reference sequences (AY179605 for NbACT deposited in GenBank). |
| qPCR- Nbactin-R | ATCAGCAATGCCCGGGAACA |  |
| VSR4-AIRAAA-R | ttaggcacgttcatcattcgtgtggttcgggacctcaggttggctgtccagtggcatgtactgtgcCGCTGCggctctgatCGCtgagtccatgtattgc | For overlapping PCR to generate *AtVSR4* mutants. |
| VSR4-AMPA-R | ttaggcacgttcatcattcgtgtggttcgggacctcaggttggctgtcCGCtggcatAGCctgtgccattatggctc |  |
| VSR4-DM-R | ttaggcacgttcatcattcgtgtggttcgggacctcaggttggctgtcCGCtggcatAGCctgtgcCGCTGCggctctgatCGCtgagtccatgtattgc |  |
| VSR4-148N/Q-R | tCTGctcgatatactttgcag |  |
| VSR4-148N/Q-F | ctgcaaagtatatcgagCAGattactataccttctgc |  |
| VSR4-294N/Q-R | cTTGcgccaccttgtaaacac |  |
| VSR4-294N/Q-F | ctttgtgtttacaaggtggcgCAAgaaaccggcaaaccttg |  |
| VSR4-434N/Q-R | tCTGggctgatttatcttgc |  |
| VSR4-434N/Q-F | gcaagataaatcagccCAGataactgcttgcaaggatac |  |
| AtVSR4-licF | CgACgACAAgACCgTCACCatgaagcagcttctatgttatct | Primers are designed for Ligation-independent cloning |
| AtVSR4-licR | gAggAgAagAgCCgTcgggcacgttcatcattcgtgtg |  |
| AtVSR4_stop_-licR | gAggAgAagAgCCgTcgttaggcacgttcatcattcgtgtg |  |
| AtVSR4-C1A-R1 | TGCAGCTGCCGCAGCcctcaatctatatttg | For overlapping PCR to generate *AtVSR4* C1A mutant |
| AtVSR4-C1A-F1 | caaatatagattgaggGCTGCGGCAGCTGCAgagatcagagccataatg |  |
