# Supplementary figures and images for: Turnip mosaic virus co-opts the vacuolar sorting receptor VSR4 to promote viral genome replication in plants by targeting viral replication vesicles to the endosome
Source: PLoS Pathog. 2022 Jan 24;18(1):e1010257. doi: 10.1371/journal.ppat.1010257 (PMC8812904; doi:10.1371/journal.ppat.1010257)

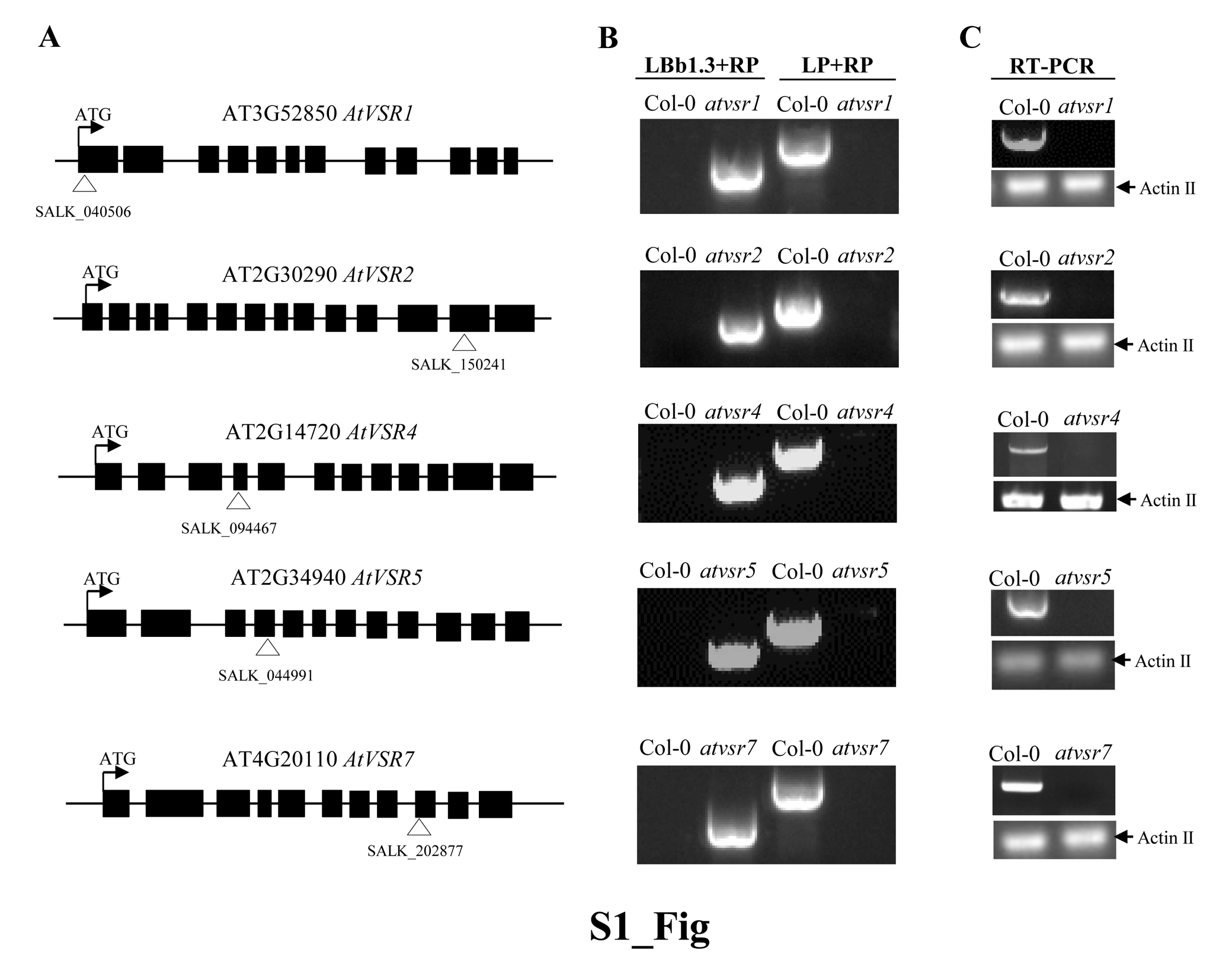

Supplement: S1 Fig — (A) Schematic diagram of the AtVSRs gene structure. The relative position of the T-DNA insertion site is shown. Exons are represented by black boxes. (B) PCR genotyping of the atvsr mutants. (C) RT-qPCR assay of the mRNA expression level of AtVSRs in the corresponding mutants. (TIF) [file ppat.1010257.s002.tif]

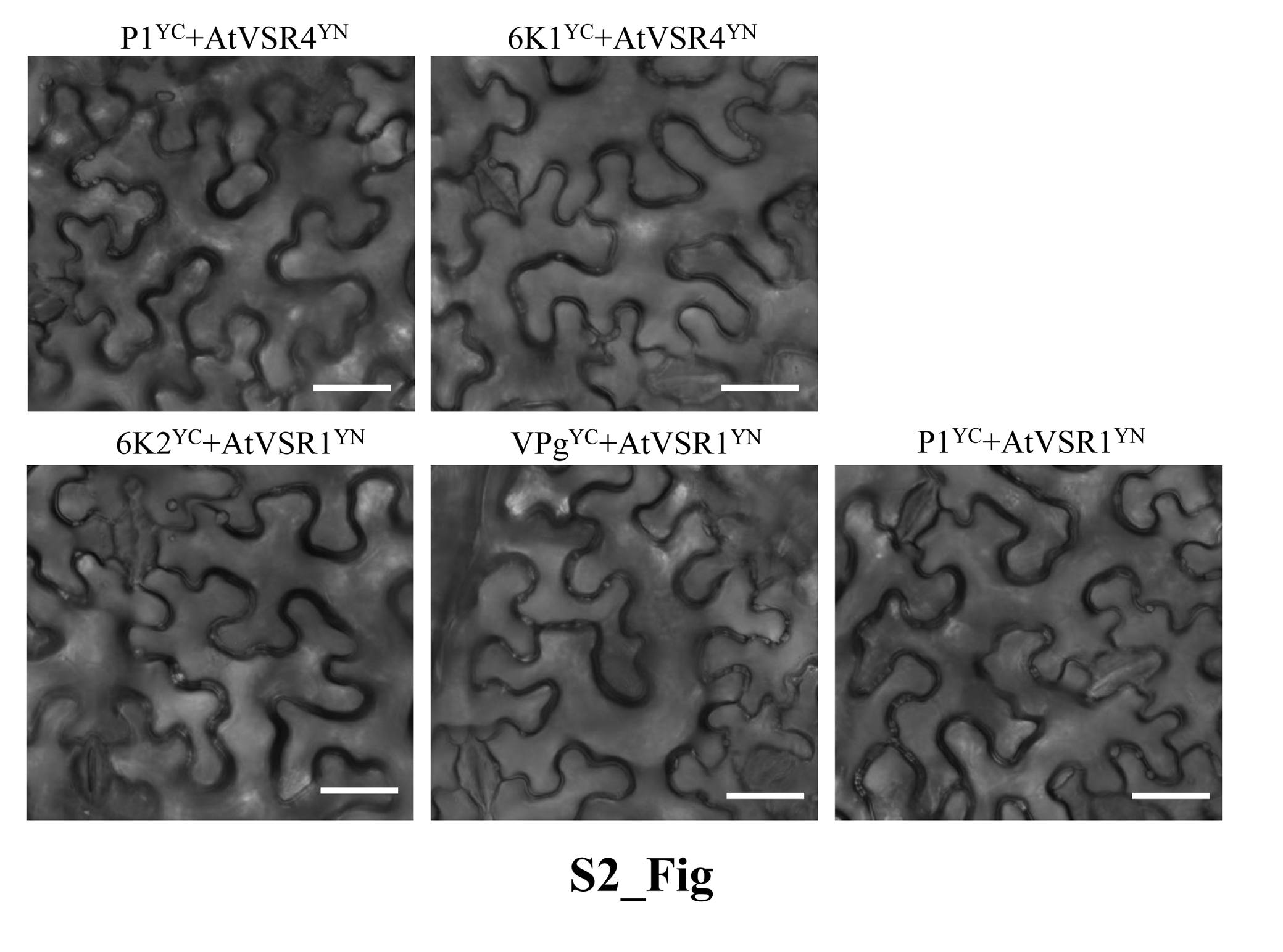

Supplement: S2 Fig — Endosome-localized AtVSR1 and TuMV-encoded membrane protein 6K1 are included as negative controls. Scale bar = 20 μm. (TIF) [file ppat.1010257.s003.tif]

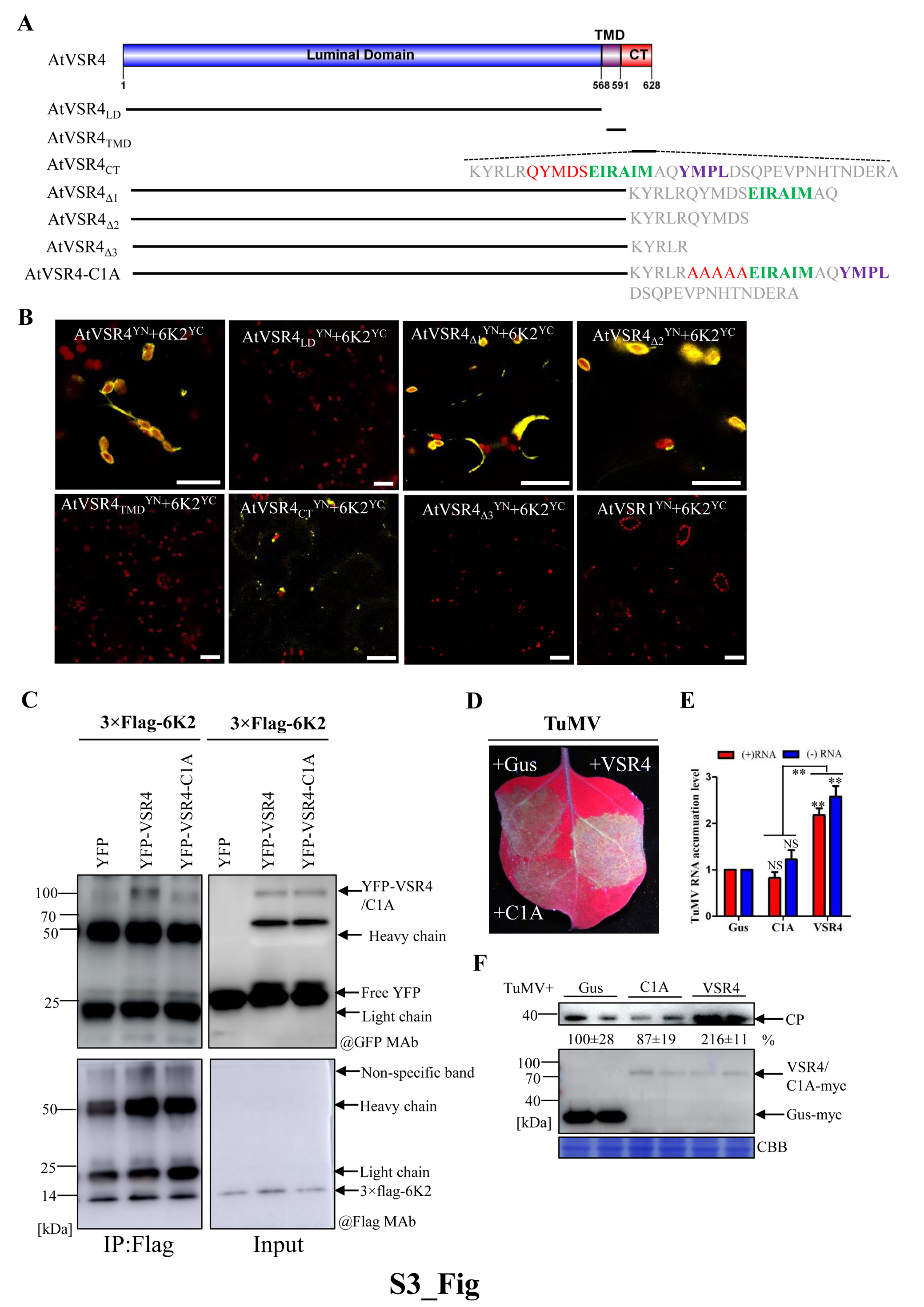

Supplement: S3 Fig — (A) Schematic representation of the functional domains of AtVSR4. Luminal domain, the N-terminal luminal binding domain; TMD, the transmembrane domain; CT, the C-terminus cytoplasmic tail. (B) Protein-protein interactions between each domain or truncated mutants in CT and TuMV 6K2 were examined in planta by BiFC. Interaction assays were performed in epidermal cells of N. benthamiana leaves. Reconstructed YFP signals were observed at 2 dpai. Scale bar = 20 μm. (C) Results of co-IP assay showing that AtVSR4-C1A cannot form complexes with TuMV 6K2 in N. benthamiana cells. Different cell lysates were immunoprecipitated with anti-Flag M2 gel beads, separated by SDS-PAGE and immunoblotted with anti-Flag monoclonal antibody (@Flag MAb), or anti-GFP monoclonal antibody (@GFP MAb). (D) GFP fluorescence in plants inoculated with TuMV-GFP together with GUS (control), AtVSR4, or AtVSR4-C1A. Plants were photographed under a hand-held UV lamp at 3 dpai. (E), Results of qRT-PCR to quantify the levels of positive-strand viral genomic RNA [(+)RNA] and negative-strand viral genomic RNA [(-)RNA] in N. benthamiana plants agroinfiltrated with different combinations of plasmids from (D). Statistical analysis was performed using Student’s t test (**, P < 0.01; NS, not significant). (F) Immunoblotting analysis of the accumulated TuMV CP levels in the infiltrated leaf tissues from N. benthamiana plants in (D) at 3 dpai. (TIF) [file ppat.1010257.s004.tif]

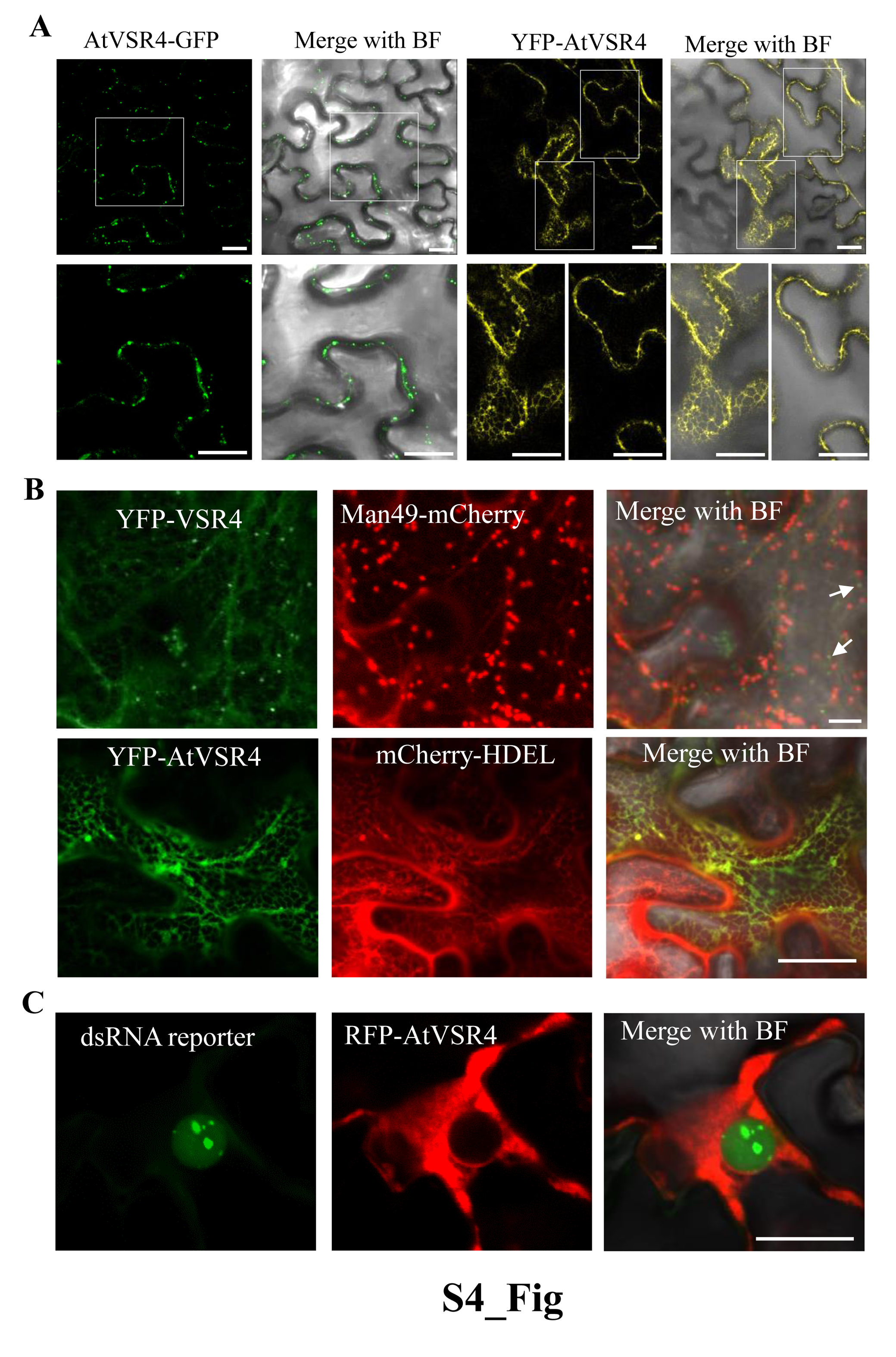

Supplement: S4 Fig — (A) AtVSR4 was fused with either GFP at the C-terminus or with YFP at the N-terminus. (B) Colocalization of AtVSR4 with a cis-Golgi marker Man49-mCherry (upper channels) or an ER marker mCherry-HDEL (lower channels). (C) Subcellular localization of RFP-AtVSR4 in 35S:B2-GFP N. benthamiana plants. Photos were taken at 2 dpai. Scale bar = 20 μm. BF, bright field. (TIF) [file ppat.1010257.s005.tif]

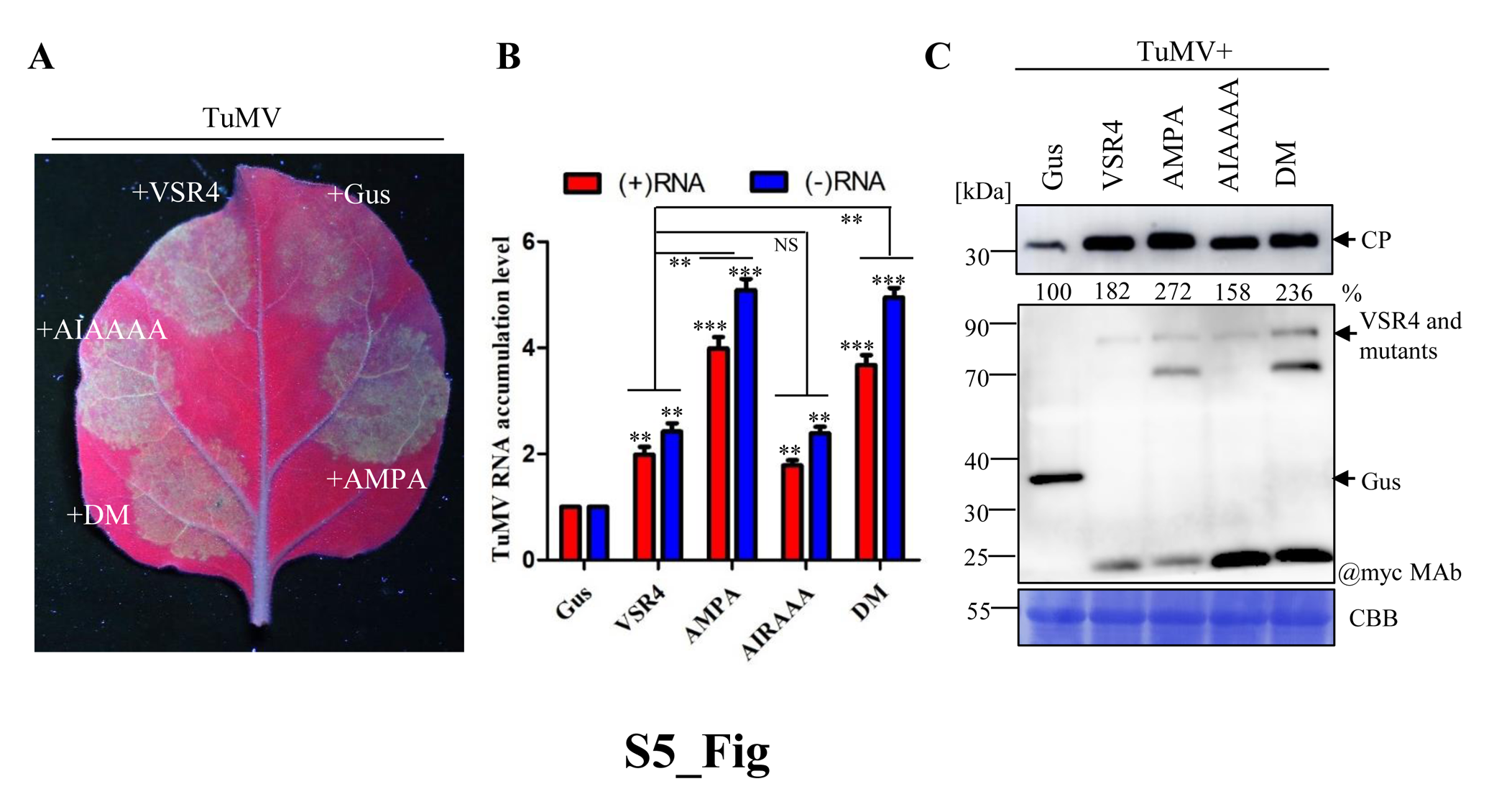

Supplement: S5 Fig — (A) GFP fluorescence in plants inoculated with TuMV-GFP together with GUS (control), AtVSR4, or its mutants. Plants were photographed under a hand-held UV lamp at 3 dpai. (B) Results of qRT-PCR to quantify the levels of positive-strand viral genomic RNA [(+)RNA] and negative-strand viral genomic RNA [(-)RNA] in N. benthamiana plants agroinfiltrated with different combinations of plasmids from (A). Statistical analysis was performed using Student’s t test (***, P < 0.001; **, P < 0.01; NS, not significant). (C) Immunoblotting analysis of the accumulated TuMV CP levels in the infiltrated leaf tissues from N. benthamiana plants in (A) at 72 hpai. (TIF) [file ppat.1010257.s006.tif]

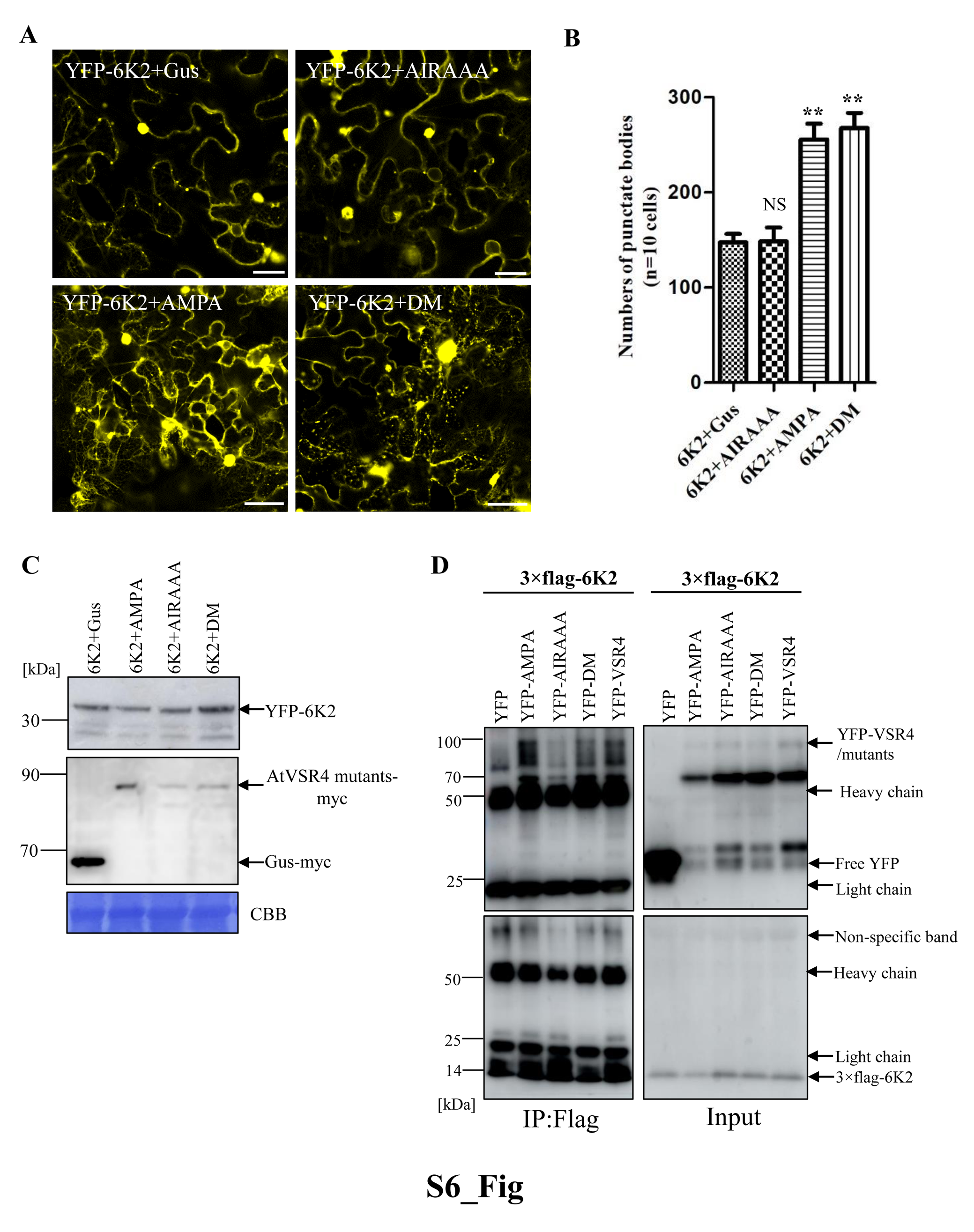

Supplement: S6 Fig — (A) Subcellular localization of YFP-6K2 when co-expressed with GUS control or with AtVSR4 mutants fused with a c-myc tag at the C-terminus in N. benthamiana cells. Scale bar = 20 μm. (B) Number of 6K2-induced punctate bodies in the cytoplasm when YFP-CI was co-expressed with AtVSR4 mutants or with the GUS control (10 cells per construct were investigated at 2 dpai and the number was calculated using Image J software). Values represent the mean number of punctate bodies ±SD per 10 cells from three independent experiments. Statistical analysis was performed using Student’s t test (**, P < 0.01; NS, not significant). (C) Immunoblotting analysis of the expression of YFP-6K2 and AtVSR4 mutants from A in N. benthamiana cells at 2 dpai. Coomassie Brilliant Blue R-250-stained RuBisco large subunit serves as a loading control. 6K2 and AtVSR4 mutants or GUS were detected with anti-GFP and anti-c-myc monoclonal antibodies, respectively. (D) co-IP assay of protein-protein interaction between 6K2 and each of the VSR4 mutants in N. benthamiana cells. Different cell lysates were immunoprecipitated with anti-Flag M2 gel beads, separated by SDS-PAGE and immunoblotted with anti-Flag monoclonal antibody (@Flag MAb), or anti-GFP monoclonal antibody (@GFP MAb). (TIF) [file ppat.1010257.s007.tif]

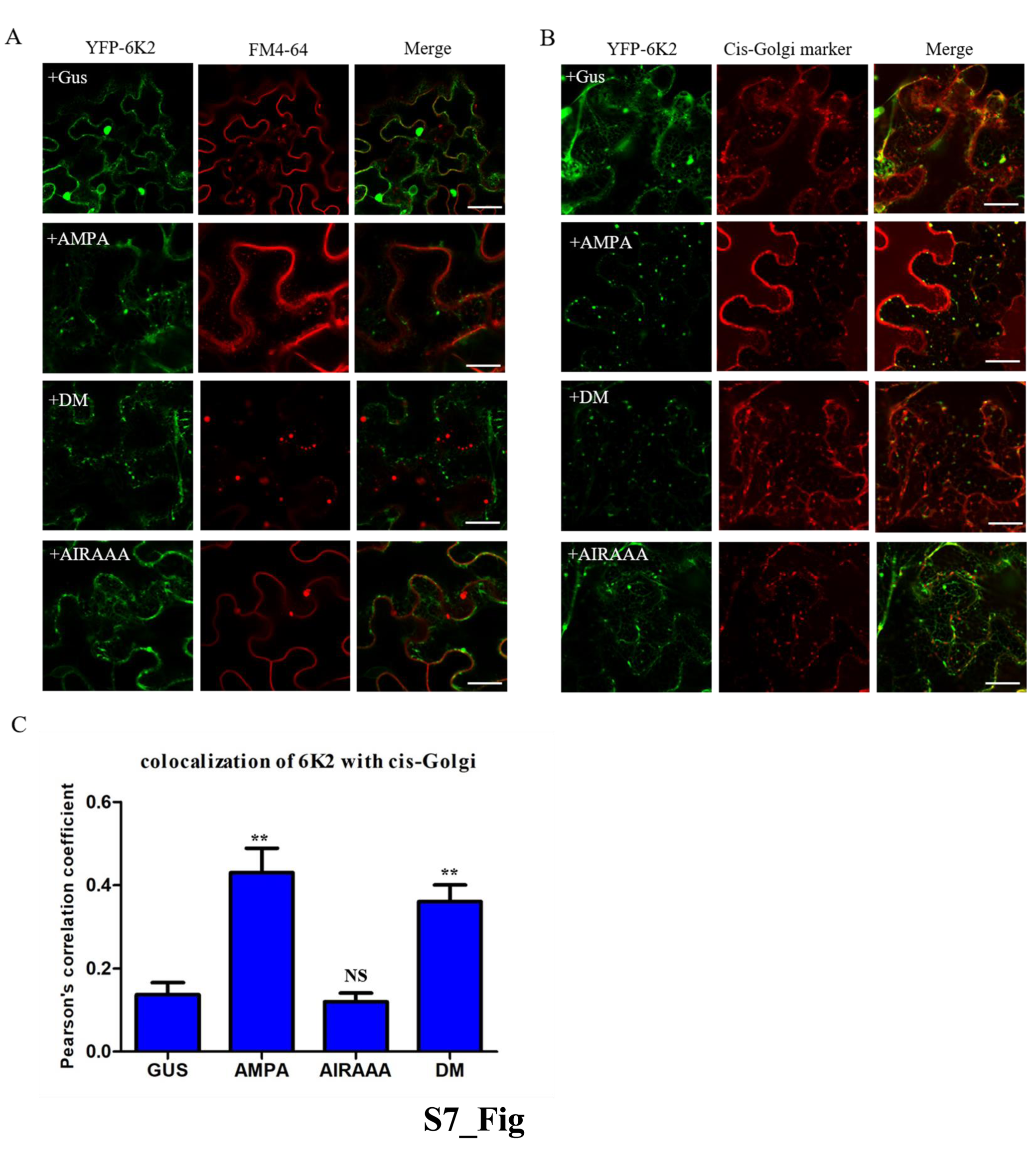

Supplement: S7 Fig — (A) colocalization of the YFP-6K2 with FM4-64. (B) colocalization of the YFP-6K2 with cis-Golgi marker (Man49-mCherry). Pictures were taken at 2 dpai. Scale bar = 20 μm. (C) Pearson’s correlation coefficient (PCC) values quantifying the colocalization between 6K2 and cis-Golgi marker under different treatments. PCC was measured from 40 cells. Error bars represent the standard deviations (SD) from three experiments. Statistical analysis was performed using Student’s t test (**, P < 0.01; NS, not significant). (TIF) [file ppat.1010257.s008.tif]

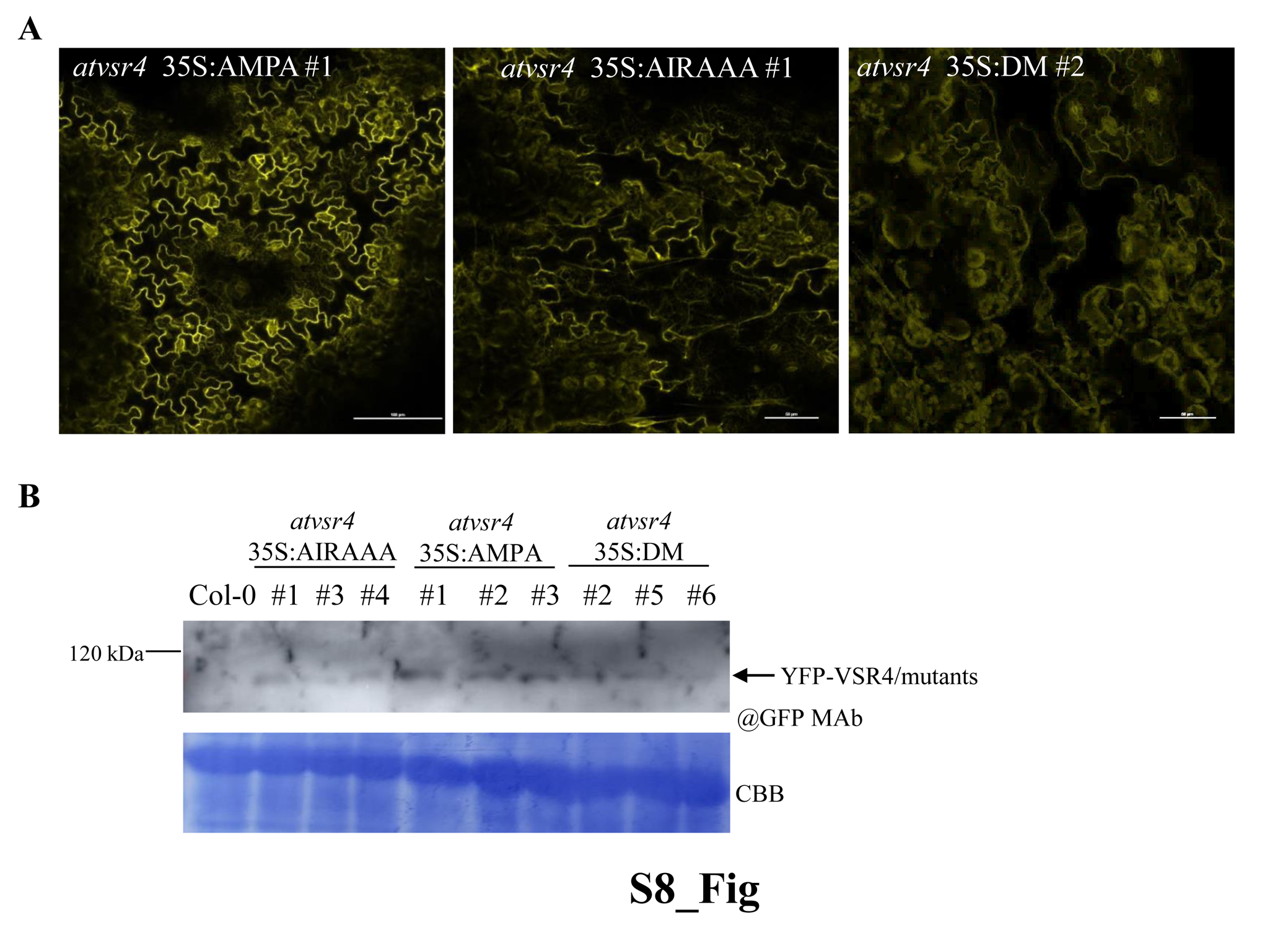

Supplement: S8 Fig — (A) Confocal examination of YFP expression in transgenic plants overexpressing different AtVSR4 mutants. Scale bar = 50 or 100 μm. (B) Immunoblotting of three positive T0 independent lines showing correct overexpression for each of three AtVSR4 mutants. (TIF) [file ppat.1010257.s009.tif]

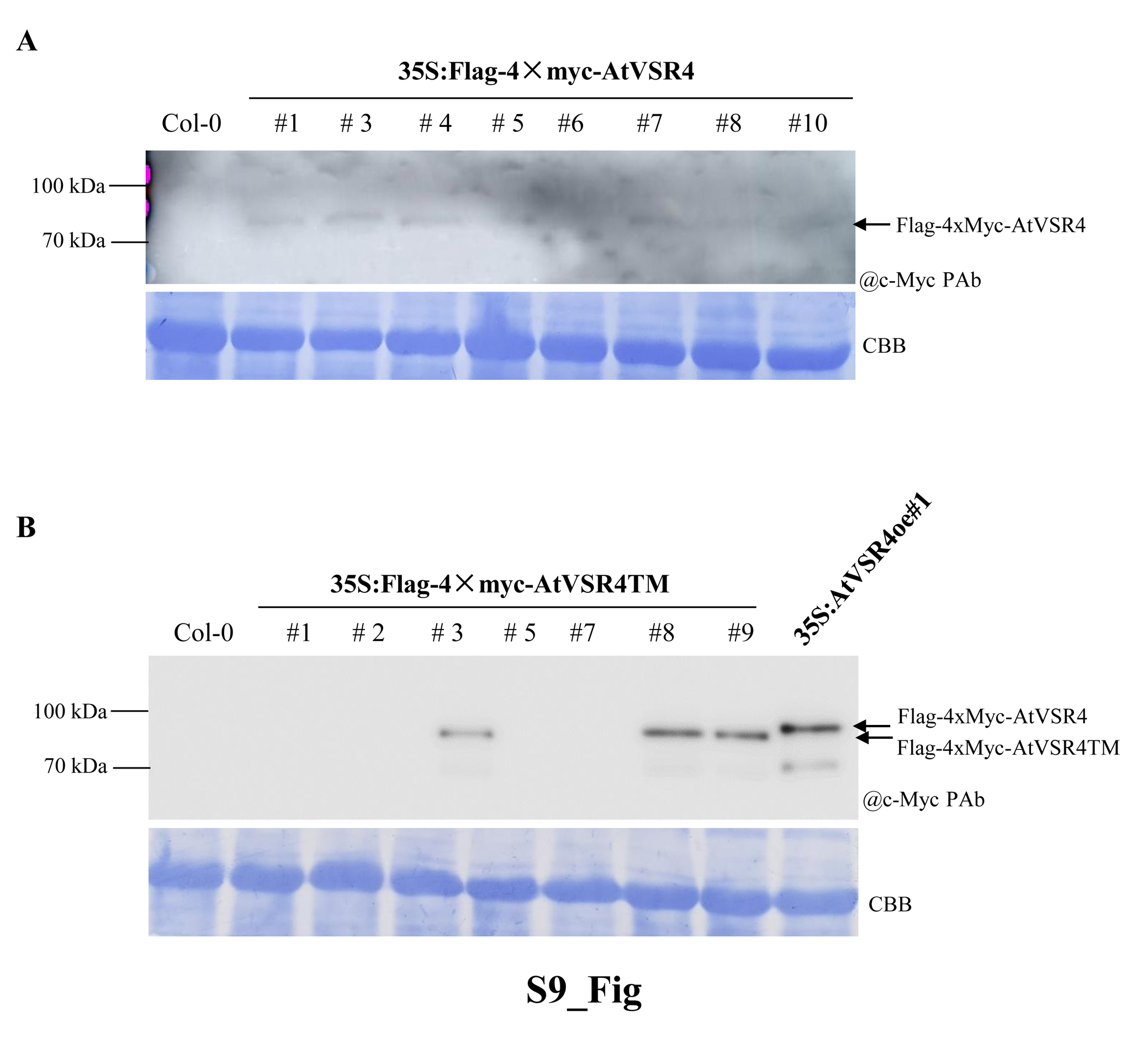

Supplement: S9 Fig — Verification of transgenic Arabidopsis lines expressing AtVSR4 (A) or TM (B). Total leaf protein extracts were immunoblotted with anti-c-myc polyclonal antibodies. (TIF) [file ppat.1010257.s010.tif]

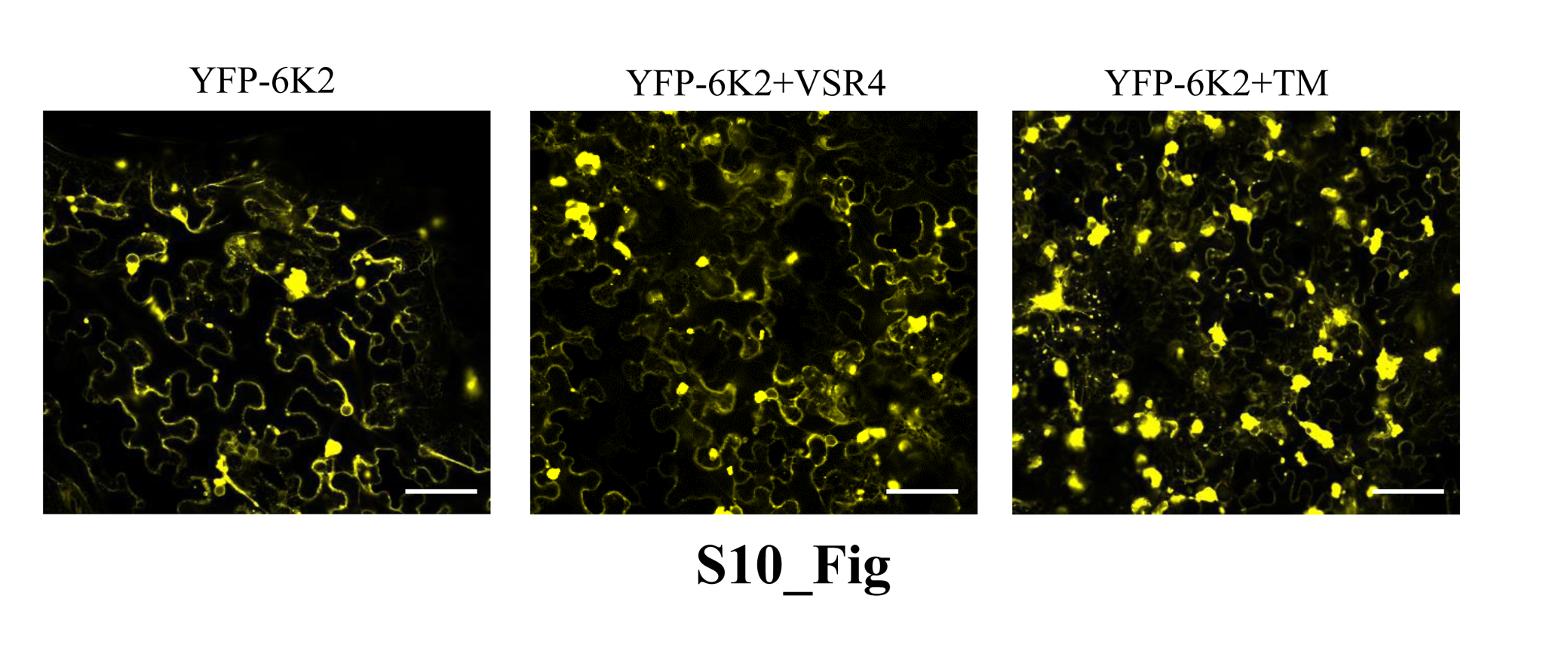

Supplement: S10 Fig — Pictures were taken at 3 dpai. Scale bar = 20 μm. (TIF) [file ppat.1010257.s011.tif]

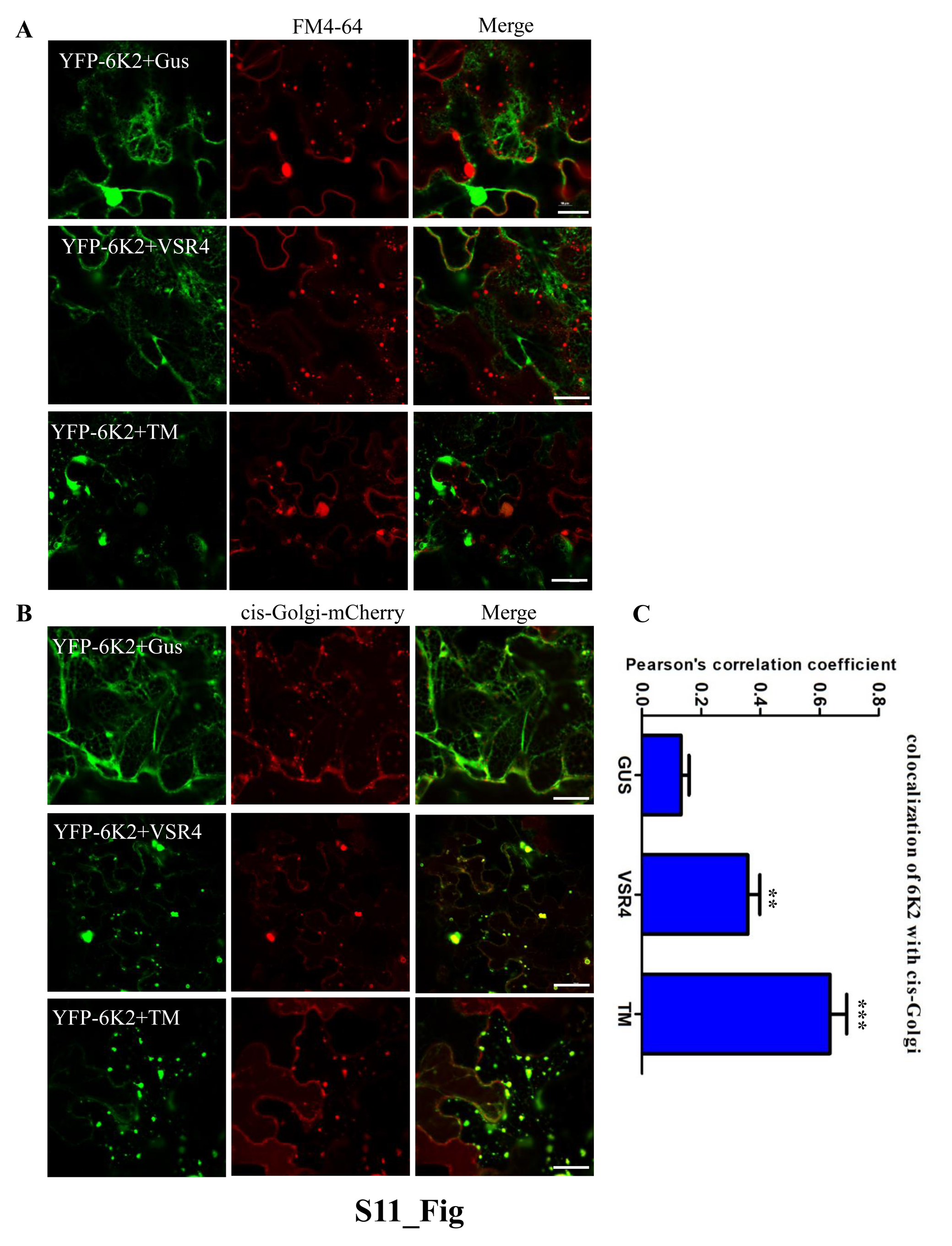

Supplement: S11 Fig — (A) Co-localization of YFP-6K2 co-expressed with either AtVSR4 or TM with FM4-64. (B) Co-localization of YFP-6K2 co-expressed with either AtVSR4 or TM with cis-Golgi marker (Man49-mCherry). Pictures were taken at 2 dpai. Scale bar = 20 μm. (C) Pearson’s correlation coefficient (PCC) values quantifying the colocalization between 6K2 and cis-Golgi maker under different treatments. PCC was measured from 40 cells. Error bars represent the standard deviations (SD) from three experiments. Statistical analysis was performed using Student’s t test (**, P < 0.01; NS, not significant). (TIF) [file ppat.1010257.s012.tif]

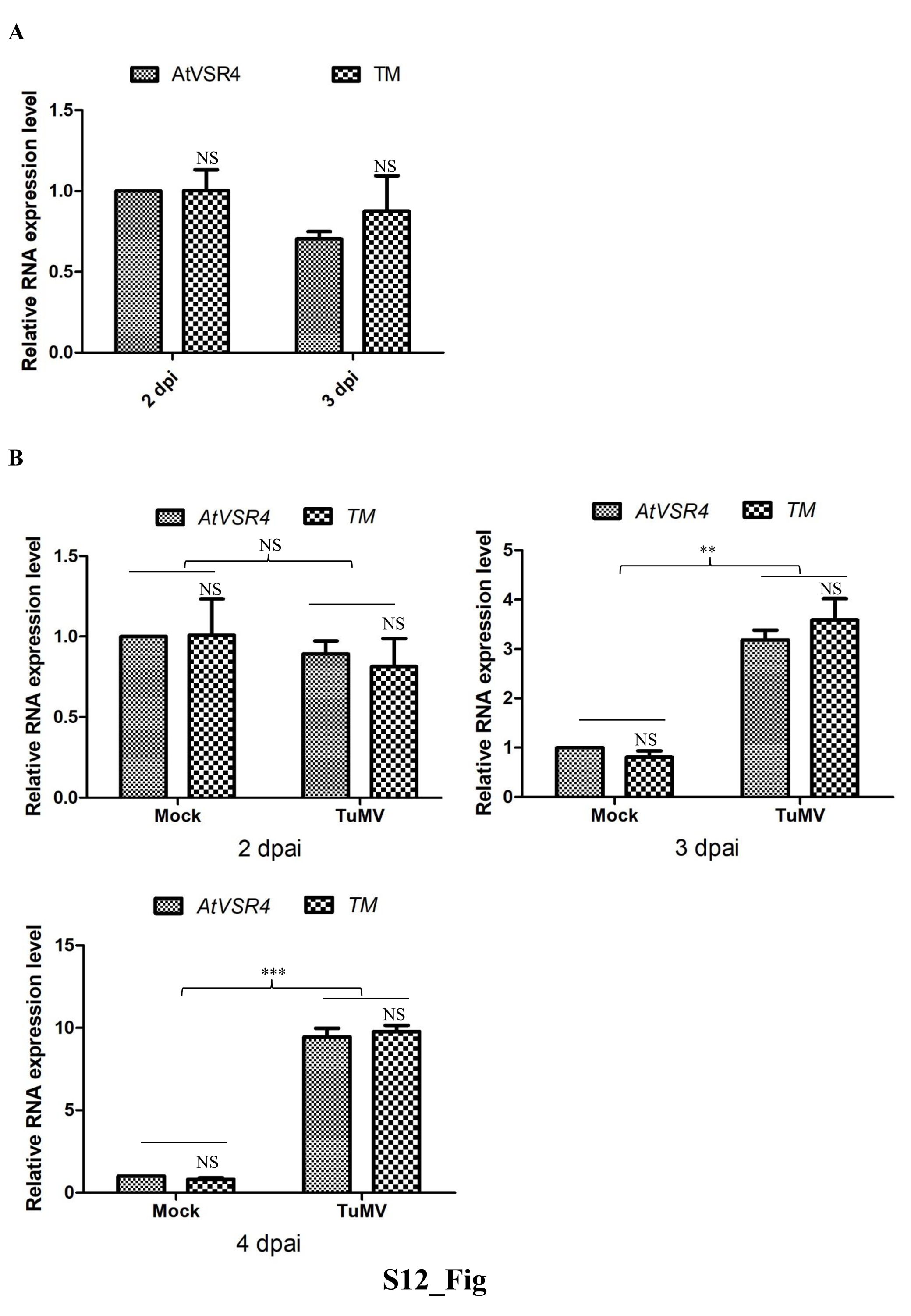

Supplement: S12 Fig — RT-qPCR analysis of mRNA levels of AtVSR4 and AtVSR4TM in non-infected controls (A) and following TuMV infection (B) at 2, 3 and 4 dpai. Error bars represent the standard deviations (SD) of three experiments. Statistical analysis was performed using Student’s t test (**, P < 0.01; ***, P < 0.001; NS, not significant). (TIF) [file ppat.1010257.s013.tif]
