# Supplementary material for: Turnip mosaic virus co-opts the vacuolar sorting receptor VSR4 to promote viral genome replication in plants by targeting viral replication vesicles to the endosome
Source: PLoS Pathog. 2022 Jan 24;18(1):e1010257. doi: 10.1371/journal.ppat.1010257 (PMC8812904; doi:10.1371/journal.ppat.1010257)
Supplement: Attachment — Submitted filename: 2021.11 responses to reviewers.docx [file ppat.1010257.s014.docx]

**Point-by-point Responses to Reviewers’ Comments**

We would like to thank the reviewers for their careful assessment of our manuscript and their suggestions, which we believe have contributed to improving both content and form. Following the reviewers’ advice, we have now made a number of changes to our manuscript, including the addition of new experimental data further supporting our conclusions; a point-by-point response to the reviewers’ comments can be found below. (Please note that line numbers, where indicated, refer to the clean version of the manuscript).

**Reviewers (Comments for the Author):**

**Part I - Summary**
Reviewer #1: PPATHOGENS-D-21-01309 reports on the identification and characterization of vacuolar sorting receptor 4 (VSR4) in trafficking of TuMV replication vesicles. VSR4 interacts with TuMV 6K2 protein and is required for TuMV to establish infection. When VSR4 is N-glycosylated, it is more stable and is more efficient at trafficking viral replication vesicles to the late endosome. N-glycosylation protects VSR4 from degradation by autophagy. The results are clear and informative.
MAIN CONTRIBUTIONS
1. A genetic analysis of five isoforms showed that VSR4 is required for TuMV infection. Single mutant plants lacking VSR4 accumulated TuMV to reduced levels and showed mild symptoms.
2. A split-ubiquitin membrane-based yeast two hybrid analysis showed that VSR4 interacts with TuMV 6K2 protein.
3. VSR4 localizes to endosomes and is recruited into 6K2-induced viral replication vesicles.
4. VSR4 is N-glycosylated, it is more stable and is more efficient at trafficking viral replication vesicles to the late endosome. N-glycosylation protects VSR4 from degradation by autophagy.

Reviewer #2: The authors report a host protein AtVSR4 which interacts with 6K2 and is involved in TuMV infection. TuMV hijacks N-glycosylated VSR4 for infection and protects VSR4 from degradation via the autophagy pathway. Some finding are interesting and the manuscript is easy to read.

Reviewer #3: This paper has shown that among the TuMV potyvirus 11 proteins, 6K2 and Vpg replication proteins interact with a vacuolar sorting receptor (VSR4). VSR4 is coopted into the TuMV VRC that likely forms on the Late endosomes. Additionally, VSR4 mutants defective in trafficking or glycosylation supported, surprisingly, higher TuMV replication than the wild type VSR4. Atvsr4 mutant plant supported moderately less TuMV replication, indicating pro-viral role for VSR4. The authors suggest that TuMV hijacks VSR4 pathway to target 6K2 vesicles to the site of replication. I have major concerns with the data and interpretation.
**Response:** We appreciate the constructive comments from the reviewers and have now added the requested experiments and revised the manuscript accordingly.
--------------------
**Part II – Major Issues: Key Experiments Required for Acceptance**
Reviewer #1: No major issues.
Reviewer #2: My major concerns about some important results and conclusions are listed below.
1. The last destination of both AtVSR4 and viral 6K2 is PVC/MVB/LE. TuMV also targets endosome for replication. And AtVSR4 is a host protein required for TuMV infection. However, overexpression of AtVSR4 mutant AMPA which disrupted the anterograde from TGN to PVC enhance virus replication. These results are contradictory.

**Response:** We apologize because it seems that our unclear description has led to some misunderstanding. TGN to LE is a very complex process and may include endocytic proteins- and VSR-dependent pathways. The former is relatively better characterized, whereas the latter still largely remain unknown. 6K2 does not hijack the canonical TGN to LE pathway mediated by endocytosis (Wu et al. 2018, Journal of Virology; Agbeci et al. 2013, PLoS Pathogens), and we therefore undertook this project to investigate the VSR-mediated TGN to LE pathway. We found that 6K2 also does not hijack the canonical TGN to LE pathway mediated by VSR proteins. Instead, 6K2 co-opts VSR4 to dissociate from cis-Golgi and directly target enlarged LE for robust replication. Overexpression of the AtVSR4 mutant AMPA can disrupt the anterograde from TGN to PVC, thus causing overaccumulation of itself at cis-Golgi, and in turn stimulating the 6K2-VSR complex to disassociate from cis-Golgi and further target enlarged LE directly for robust replication, as indicated in Fig 9. We have added what we hope is a clearer description in the text (L25-27, L254-255).

2.In Fig. 3A, YFP-AtVSR4 did not co-localize with FM4-64 labeled endosome. They were only adjacent.
**Response:** This was our careless mistake – the arrows did not point at the right positions and we have corrected these in this version.

3. In Fig. 3C, TuMV::6K2-mCherry didn’t co-localize with AtVSR4-GFP and it seems like be in different layer. And, RFP-AtVSR4 didn’t co-localize with dsRNA reporter because they did not merge. The green punctate bodies labelled by arrows didn’t appear in red channel. Points 3 and 4 are key to the final conclusion.

**Response:** Following the reviewer’s suggestion, we have provided better pictures to support the conclusion that TuMV 6K2-indcuced vesicles indeed colocalize with AtVSR4-formed granules at the two time points examined (Fig 3C). In this manuscript, we provide clearer pictures, which include chloroplasts in the colocalization assay of RFP-AtVSR4 and dsRNA reporter. As shown in Fig 3D, in addition to localizing at the nucleus in the absence of viral infection (Fig S4C), dsRNA reporter signals appeared at perinuclear region under TuMV infection and merged with RFP-AtVSR4.

4.The author has found the 597QYMDS601 motif in AtVSR4 CT was responsible for binding to 6K2. Only five amino acids exist in this motif. Alanine scanning can be performed to confirm the key amino acid(s) and the resulting mutant should be used to test if its transient overexpression influence TuMV-GFP.

**Response:** Thank you for this insightful suggestion which we have followed and which has helped confirm the biological significance of the VSR4-6K2 interaction in TuMV infection. To be convenient, we mutated all five amino acids to alanine, producing AtVSR4-C1A (Fig S3A). Co-IP results then showed that this mutant indeed cannot bind with 6K2 (Fig S3C). When AtVSR4-C1A was transiently overexpressed, CP accumulation was comparable to that in the GUS control, and much less than in the AtVSR4 treatment (Fig S3D and S3E), showing that a VSR4-6K2 interaction is required for TuMV infection. We have added these new results in this manuscript (L186-192).

Reviewer #3: 1. Because it has been shown previously that 6K2 does not traffic through the canonical TGN pathway, I am not sure what is the interpretation of the VSR4 mutants affecting the two-way trafficking between TGN and LE (S5 Fig). AMPA and DM mutants are predicted to stuck in TGN, but 6K2 does not traffic through TGN. Indeed, these mutants were ignored in the model (Fig. 9). The actual subcellular localization of these mutants should be tested in TuMV-infected plants, because they greatly affect TuMV replication. Also interaction of these mutants with 6K2 and VpG should be tested. Is there TuMV replication in cis-Golgi?

**Response:** We apologize that our unclear description in this part may have resulted in some misunderstanding. Our results regarding the VSR4 mutants affecting the two-way trafficking between TGN and LE suggested that 6K2 does not hijack the canonical TGN to LE pathway mediated by VSR proteins, instead, 6K2 co-opts VSR4 to dissociate from cis-Golgi and further targets enlarged LE directly for robust replication. The accumulation of VSR4 and its mutants AMPA and DM at cis-Golgi can facilitate this progress. We have included these mutants in our revised model (Fig 9).

Following the reviewer’s advice, the subcellular localization of these VSR4 mutants in TuMV-infected plants was examined. As shown in Fig 4C, several granules formed by VSR4, AMPA and DM colocalized with TuMV 6K2-induced vesicles around chloroplasts, while fewer AIRAAA-formed granules decorated 6K2-induced vesicles (L271-274). VPg did not reliably interact with VSR4 (Fig 2) and thus we have focused our study on 6K2. These mutants all still interact with 6K2, since the mutant sites are not within the interaction motif, as shown in Figs S3A-C and S6D. However, AIRAAA had a much weaker binding capacity than wild type VSR4, AMPA and DM mutants (L262-264).

We performed dsRNA localization with cis-Golgi marker Man49-mCherry under TuMV infection, and did not find perfect colocalization (see Author response image 1 below), suggesting that Golgi may not serve as a replication site for TuMV.

Author response image 1: Colocalization of dsRNA reporter with the cis-Golgi marker Man49-mCherry under TuMV infection

2. VSR4TM mutant is defective in glycosylation, therefore VSR4TM might not traffic out of ER and it might induce ER stress; this could be the mechanism of its action of VSR4TM on TuMV replication.
**Response:** We thank the reviewer for this helpful comment. We cannot exclude the possibility that ER stress is involved in TuMV infection but ER stress has actually been reported to inhibit TuMV infection (Gaguancela et al. The IRE1/bZIP60 Pathway and Bax Inhibitor 1 Suppress Systemic Accumulation of Potyviruses and Potexviruses in Arabidopsis and Nicotiana benthamiana Plants, 2016, MPMI; Gayral et al. Multiple ER-to-nucleus stress signaling pathways are activated during Plantago asiatica mosaic virus and Turnip mosaic virus infection in Arabidopsis thaliana, 2020, The plant Journal). We also used tunicamycin treatment to induce ER stress and indeed found that ER stress inhibits TuMV infection (see our new results for review in the point 3 below). Thus, we believe that ER stress is unlikely to account for the positive role of VSR4TM in TuMV replication.

3. I am not convinced that VSR4 plays a direct role as claimed in Fig. 9 based on VSR4 mutants. I think the role of ER or Golgi stress caused by expression of VSR4 mutants might be involved in stimulating TuMV replication and 6K2 body formation. For example, Tunicamycin treatment inhibits glycosylation of VSR4, similar to the TM mutant. It is then predicted that Tunicamycin treatment might also enhance TuMV replication? The interaction of VSR4 mutants and 6K2 has not been tested rigorously in this work (see also below).
**Response:** Following the reviewer’s helpful suggestion, we performed additional experiments that show that VSR4-6K2 interaction is critical for TuMV infection (Fig S3). We constructed a VSR4 mutant, namely VSR4-C1A, which introduced alanine to replace the interaction motif ^597^QYMDS^601^ (Fig S3A and S3B). Co-IP results shown that this mutant indeed cannot bind with 6K2 (Fig S3C). Transient overexpression of AtVSR4-C1A showed comparable CP accumulation to that in the GUS control, but much less than in the AtVSR4 treatment (Fig S3D and S3E). We have added these new results in this manuscript (Fig S3) (L186-192).

Regarding the role of ER or Golgi stress may caused by VSR and mutants in TuMV infection, yes, it would be interesting to study if VSR4 and mutants would affect ER or Golgi stress and further TuMV infection. ER stress has been reported to inhibit TuMV infection (Gaguancela et al. 2016, MPMI; Gayral et al. 2020, The plant Journal), thus, it seems to be unlikely caused from ER stress. Following this reviewer’s advice, we used tunicamycin treatment to induce ER stress and analyzed the effect of ER stress on TuMV infection. As shown in Author response image 2 below, Tunicamycin treatment supressed TuMV infection. This result is also consistent with previous reports (Gaguancela et al. 2016, MPMI; Gayral et al. 2020, The plant Journal). Thus, we believe that ER stress is unlikely to provide a mechanism for the positive role of VSR4TM on TuMV replication.

Author response image 2: Effect of tunicamycin treatment on TuMV infection in *N*. *benthamiana* cells.

Plants were photographed under a hand-held UV lamp at 3 dpai (A). TuMV CP was detected with anti-TuMV CP polyclonal antibody at 3 dpai (B).

Information about Golgi stress in plants is currently very limited and it is difficult to determine what role this might play. To properly answer this question would need a number of experiments. However, we believe that we have confirmed the critical role of the VSR4-6K2 interaction in TuMV infection in this new version, suggesting that VSR4 plays a direct role in TuMV infection. As this study mainly focuses on the biological significance of VSR-TuMV interaction and contains a large volume of data, we prefer not to expand it further. We agree that Golgi stress possibly caused by over-accumulation of VSR and mutants in cis-Golgi might contribute to TuMV infection, and have acknowledged this in the discussion (L508-510).

Following the reviewer’s suggestion, the interaction of VSR mutants and 6K2 has been examined and added in this new version. (1) Co-IP results show that VSR mutants can still bind with 6K2, although AIRAAA had a much weaker binding capacity that the wild type VSR4 and the AMPA and DM mutants (Fig S6D); (2) the subcellular localization of VSR mutants with TuMV 6K2-induced vesicles is now demonstrated (Fig 4C). We have added these new results in this manuscript (L262-264, L272-274).

4. What happens to 6K2 protein localization in atvsr4 mutant plant? Why was that study omitted? If VSR4 pathway is directly involved in TuMV replication, then 6K2 is not expected to reach LE. This can be tested in atvsr4 mutant plant.
**Response:** We agree. Following the reviewer’s suggestion, we performed 6K2 subcellular localization in the protoplasts of Col-0 and *atvsr4*. Our results showed that 6K2 indeed cannot reach enlarged LE efficiently in the *atvsr4* mutant compared with that in Col-0 (see our new results shown in Fig 3E and 3F, L217-225).

5. The role of co-opted LE and the noncanonical pathway of 6K2 trafficking in TuMV replication are not novel, documented in several papers previously. The critical role of the cis-Golgi in TuMV replication in this paper has not been established unambiguously. Also, replication of TuMV was only moderately inhibited in atvsr4 mutant plant. Therefore, the paper is too preliminary at this point.
**Response:** We agree that the role of co-opted LE and the noncanonical pathway of 6K2 trafficking in TuMV replication has been demonstrated in several reports (Agbeci et al. 2013, PLoS Pathogens; Cabanillas et al. 2018, Plant Cell; Wu et al. 2020, The Plant Journal). In recent years, the importance of proteins from the ESCRT family for BMV and TBSV infection also provides some indication for the involvement of PVCs/MVBs in plant virus infection. However, information on the explicit involvement of PVCs/MVBs, especially the host factors, is still limited for other plant viruses.

Here, we focused on the host factors involved in the trafficking of replication vesicles to LE, and used an important potyvirus member TuMV as a case study. In this report, we provide evidence that TuMV replication depends on functional vesicle transport from cis-Golgi to the late endosome pathway that is mediated by a specific VSR family member VSR4 from Arabidopsis. Knock out of VSR4 impaired the targeting of TuMV replication vesicles to enlarged LE, and this process depends on the specific interaction between VSR4 and the viral replication vesicle-forming protein 6K2. We also showed that the N-glycosylation of VSR4 modulates the targeting of TuMV replication vesicles to enlarged LE and viral infection, thus contributing to our understanding of how TuMV manipulates host factors in order to establish their optimal infection.

Because our current data do not support the idea that cis-Golgi can be a replication site for TuMV but do show that VSR4 plays a direct role in TuMV infection, we prefer not to expand the paper even further (see our responses for points 1 and 3). The knockout mutants of other AtVSR members do not show significant resistance to TuMV infection, and viral accumulation levels in these mutants is similar to that in Col-0 plants (Fig 1). We cannot exclude a possible functionally redundant role for AtVSRs in TuMV infection. Knock out of two or more AtVSR member in the same plant may further identify the critical role of VSR in TuMV infection but this would be very time consuming. Finally, we identified an important VSR member AtVSR4 for TuMV infection and dissected the molecular mechanism. Thus, this work opens a new research area in plant virology since previously very little work has been done to understand the involvement of VSR in plant virus infection, and the results may have implications for the role of VSR in other positive-strand RNA viruses.

--------------------
**Part III – Minor Issues: Editorial and Data Presentation Modifications**
Please use this section for editorial suggestions as well as relatively minor modifications of existing data that would enhance clarity.

Reviewer #1: 1. Non-glycosylated VSR4 promotes higher TuMV accumulation than N-glycosylated VSR4 (Fig. 6). However, VSR4 is N-glycosylated, it is more stable and is more efficient at trafficking viral replication vesicles to the late endosome. N-glycosylation protects VSR4 from degradation by autophagy. An explanation is needed for these seemingly contradictory observations.
**Response:** Although VSR4 is *N*-glycosylated and more stable, it is much weaker at mediating the trafficking of 6K2 vesicles to enlarged LE compared with non-glycosylated VSR4 (Fig 7). It is possible that overexpression of the non-glycosylated AtVSR cannot bind with vacuolar cargoes efficiently (Shen et al. 2014, The Plant J), and therefore an increase in the accumulation of non-glycosylated VSRs in the cis-Golgi can be beneficial to TuMV infection. This idea was supported by the observation that more 6K2 punctate bodies apparently localized at cis-Golgi when AtVSR4TM was co-expressed as compared with VSR4 overexpression (Fig S11C). Actually, both N-glycosylated and non-glycosylated VSR4 go into autophagy degradation (Fig 8) but TuMV 6K2 can recruit VSR to avoid its degradation to benefit viral infection. We have added a clearer description in the text (L25-27, L254-255).

Reviewer #2: (No Response)
Reviewer #3: L158: The localization of VSR4 and 6K2 to chloroplasts is ignored in follow up experiments. What is the reason to focus only on LE?

**Response:** We thank the reviewer for pointing this out. 6K2 alone can localizes to chloroplasts, and it is therefore reasonable that the VSR4-6K2 complex can also target chloroplasts. In this study, we aim to investigate how 6K2 targets LE, and whether VSRs participate in this progress. Thus, we have focused on the biological significance of the 6K2-VSR4 complex in LE in follow up results. We have added an explanation (L176-179).

L194 and L198: There is no endosomal marker shown in Fig. 3B. Therefore, I am not sure that it is correct to claim that “VSR4-labeled endosomes colocalized…” Indeed, VSR4 was shown by the authors to localize to several compartments, not only LE.
**Response:** Following the reviewer’s suggestion, we performed new experiments adding an endosomal marker to show that VSR4-labelled punctate bodies colocalized with YFP-6K2 and FM4-64 stained enlarged LE (Fig 3B).

L267: Why was atvsr4 plant (not expressing the mutants) not used in this complementation assay as a control?
**Response:** Yes, it would be much better as the reviewer pointed out, since these mutants were overexpressed in the *atvsr4* background. Here, we aimed to investigate the effect of AtVSR4 mutants’ overexpression in *atvsr4* background compared with AtVSR4 on TuMV infection. Because Col-0 has endogenous AtVSR4, we have used col-0 as the control.

L298-308: references on the enzymes’ activities are missing here.
**Response:** We thank the reviewer for pointing out this omission; this information has now

been added to the manuscript (L317).

Fig. 2C The coIP is low quality and not convincing. What are the 3 bands in the co-IP lane for VSR4? Only 1 faint band is seen in input.
**Response:** We have now repeated the co-IP experiment and present clearer blots in Fig 2C.

Fig. 2D: The localization of the Golgi marker seems to be mostly cytosolic in the shown image! Please look at S4B fig for Golgi localization. The distribution of the ER marker is also unusual. Why were those merged with BF images, and the FM4-64 image was not merged with BF?

**Response:** Golgi stacks can be found throughout the cells as small, nearly round spots. Here, we used Man49-mCherry as a cis-Golgi marker (Nelson et al. 2007, The Plant Journal), and can see typical cis-Golgi-like structures, indicating that this marker indeed works. We have provided better pictures in this version of the manuscript to show the distribution of the ER marker mCherry-HDEL. There is also a better FM4-64 image. These new pictures do not affect our conclusions.

Fig. 3A: the two VSR4 images are incredibly different: what is the reason?
**Response:** VSR4 can be observed at multiple sites in plant cells, including ER-like and endosome-like structures as shown in Fig S4A. Thus, different layers of plant cells have been captured to further confirm the localization of VSR4 to endosome and ER with corresponding markers. We have improved the images and our conclusion is not affected.

Fig. 3C dsRNA analysis: I am not convinced that there is co-localization between dsRNA and VSR4 based on the shown image. Most of the GFP signal is far away from the VSR4 signal.
**Response:** We are sorry for our careless mistake. The arrows were incorrectly positioned. In this manuscript, we include chloroplasts in the colocalization assay of RFP-AtVSR4 and dsRNA reporter and provide clearer pictures. As shown in our new Fig 3D, in addition to being localized at the nucleus in the absence of viral infection (Fig S4C), dsRNA reporter signals appeared in the perinuclear region under TuMV infection and merged with RFP-AtVSR4.

Fig. 8A-B: TM-myc (and possibly TM-nLuc) is so poorly expressed in plants (barely detectable) that it is not fair to compare it with VSR4-myc: the authors’ claim of weakened interaction of TM with 6K2 when compared with VSR4 is not justified.
**Response:** We agree. We have revised this conclusion in this new version but the results still indicate that AtVSR4TM can interact with 6K2 (see L331-334).

Fig. S4 B-C: How can VSR4 subcellular distribution be so different in the two images?
**Response:** VSR4 can be observed at multiple sites in plant cells, including ER-like and endosome-like structures.RFP-AtVSR4 showed similar localizations to that of YFP-AtVSR4. In addition to forming punctate bodies along the PM, RFP-VSR4 also showed some aggregates around the nucleus, which are believed to be the ER localization (see Author response image 3 below). We have zoomed into a specific region to show its colocalization with different organelle markers.

Author response image 3: Subcellular localization of RFP-AtVSR4 in *N*. *benthamiana* cells.

L 334 and Fig. 6D. “much higher fluorescence intensity” with the TM mutant is not obvious based on the image shown.
**Response:** Following the reviewer’s suggestion, since TMoe showed comparable fluorescent intensity with VSR4oe, we have revised the text with “At 8 dpi, both AtVSR4TMoe and AtVSR4 showed a remarkably higher intensity of GFP fluorescence than Col-0 plants…..” (L354-355).

S11B Fig. The distribution of cis-Golgi marker and also 6K2 changed when VSR4 and TM were expressed. Explanation?
**Response:** Membrane trafficking pathways play pivotal toles in rapid responses to environmental stimuli in plants (Inada and Ueda, Membrane Trafficking Pathways and their Roles in Plant–Microbe Interactions, 2014, Plant Cell & Environment). We suppose that VSR4 and TM may affect ER or Golgi homeostasis and further secretory vesicles trafficking, and so we can observe the change of 6K2 distribution. Golgi stacks can be found throughout the cells as small, nearly round spots, as shown in Fig S11B. This observed phenomenon is consistent with our conclusion.

L498: What makes LE “enlarged” in the model?
**Response:** Live cell imaging and EM analysis suggested that these spherical structures, i.e. enlarged LE, are derived largely from the homotypic fusion of MVBs (Jia et al. ARA7(Q69L) expression in transgenic Arabidopsis cells induces the formation of enlarged multivesicular bodies. 2013, Journal of Experimental Botany). Currently we do not know whether VSR participates in this progress, which needs further investigation. Previously we omitted enlarged LE from the model but we have added it in this revised manuscript.

L502-504: It is unlikely that TuMV evolved and adapted to hosts used in this work: N. benthamiana or Arabidopsis, in nature. Therefore, I do not think this part of the discussion on VSR4 glycosylation and TuMV evolution has a merit.
**Response:** As suggested, this part has now been deleted.
