# Supplementary material for: Turnip mosaic virus co-opts the vacuolar sorting receptor VSR4 to promote viral genome replication in plants by targeting viral replication vesicles to the endosome
Source: PLoS Pathog. 2022 Jan 24;18(1):e1010257. doi: 10.1371/journal.ppat.1010257 (PMC8812904; doi:10.1371/journal.ppat.1010257)
Supplement: Attachment — Submitted filename: Review PPATHOGENS-D-21-02305.docx [file ppat.1010257.s015.docx]

Review PPATHOGENS-D-21-02305

This manuscript addresses the identification of AtVSR4 as an interactor of TuMV 6K2 protein and aims to characterize its role in targeting 6K2 to the enlarged late endosome for efficient viral infection instead of using the conventional VSR-mediated pathway from early to late endosome. The authors report VSR4 mutants defective in trafficking and non-N-glycosylated forms that support stronger viral replication than the wild type VSR4. At the same time, the virus promotes VSR4 accumulation and hijack glycosylated VSR4 for its protection from autophagy pathway. The manuscript is original and provides a large amount of information on the role of 6K2 co-opting VSR4 and the way they are able to traffic to promote viral replication. These results are relevant for the plant-pathogens community. The methodology is adequateThe manuscript is dense to read probably because it has a large amount of information. Overall, it is well written.

The doubts I had referred to mutants and non-N-glycosylated VSR4 promoting viral infection. Then, wild type VSR4 would be somehow down controlling viral infection. However, this question has already been addressed by the authors in response to the previous reviewers and included in the new version. Thus, I have no further requirements.
