# Supplementary material for: Turnip mosaic virus co-opts the vacuolar sorting receptor VSR4 to promote viral genome replication in plants by targeting viral replication vesicles to the endosome
Source: PLoS Pathog. 2022 Jan 24;18(1):e1010257. doi: 10.1371/journal.ppat.1010257 (PMC8812904; doi:10.1371/journal.ppat.1010257)
Supplement: Attachment — Submitted filename: 2022.1.4 Responses to reviewers comments.docx [file ppat.1010257.s016.docx]

**Point-by-point Responses to Reviewers’ Comments**

We would like to thank two anonymous reviewers for their careful assessment of our manuscript and their suggestions. Following the reviewer#2’s advice, we have now made a number of changes to our manuscript, including the addition of new experimental data further supporting our conclusions; a point-by-point response to the reviewers’ comments can be found below.

**Reviewers (Comments for the Author):**

**Part I - Summary**
Reviewer #1: This manuscript addresses the identification of AtVSR4 as an interactor of TuMV 6K2 protein and aims to characterize its role in targeting 6K2 to the enlarged late endosome for efficient viral infection instead of using the conventional VSR-mediated pathway from early to late endosome. The authors report VSR4 mutants defective in trafficking and non-N-glycosylated forms that support stronger viral replication than the wild type VSR4. At the same time, the virus promotes VSR4 accumulation and hijack glycosylated VSR4 for its protection from autophagy pathway. The manuscript is original and provides a large amount of information on the role of 6K2 co-opting VSR4 and the way they are able to traffic to promote viral replication. These results are relevant for the plant-virus community. The methodology is adequate. The manuscript is dense to read probably because it has a large amount of information. Overall, it is well written.

The doubts I had referred to mutants and non-N-glycosylated VSR4 promoting viral infection. Then, wild type VSR4 would be somehow down controlling viral infection. However, this question has already been addressed by the authors in response to the previous reviewers and included in the new version. Thus, I have no further requirements.

Reviewer #2: This manuscript continues recent investigations on ER-derived 6K2-induced vesicles and how they utilize an unconventional Golgi-bypassing pathway and enlarged late endosomes to induce a productive turnip mosaic virus infection. The authors show through extensive experimental work that vacuolar sorting receptor 4 (VSR4) has a specific role in this process through its interaction with the 6K2 protein. The authors demonstrate that QYMDS sequence at a C-terminal domain of VSR4 serves as a binding site and when this sequence was substituted with five alanine residues both 6K2 binding and CP accumulation were reduced (Fig. S3). All conditions that increased the amount of VSR4 and those mutations in it which prevented its normal function in Golgi traffic, enhanced 1) the amount of unconventional bypass of 6K2-vesicles from cis-Golgi to enlarged late endosomes and 2) both (+)- and (-)-strand TuMV RNA accumulation. N-glycosylation is used for the turnover regulation of VSR4. TuMV infection increased the amount of VSR4 on protein but not on mRNA level, which proposes that TuMV can affect the degradation of glycosylated VSR. The authors have done a substantial revision and added a lot of data to this revised version. I find the presented data very interesting.

**Responses:** We appreciate the constructive and positive comments from the reviewers.

**Part II – Major Issues: Key Experiments Required for Acceptance**

Reviewer #1: (No Response)

Reviewer #2: Among the added data is Fig S3 in which it is shown that QYMDS to AAAAA substitution in C-terminus of VSR inhibited the VSR4 binding to 6K2 and reduced TuMV CP accumulation. The quantitation of CP accumulation is missing from this figure. In the case of other VSR4 mutants as well as when over expressed or down regulated, the authors demonstrate viral RNA accumulation. How does OE of VSR-C1A mutant alter the (+)- and (-)-strand synthesis? I don’t agree yet with the statement that VSR4-6K2 interaction is critical for TuMV infection.

**Responses:** Thanks for this constructive suggestion. Following the reviewer’s advice, we repeated the experiment and improved the data on this part. As shown in our new prepared S3 Fig D-F, the quantitation of CP accumulation has been added, and both the (+)- and (-)-strand viral RNA accumulation in *N*. *benthamiana* cells overexpressing VSR4-C1A, wild type VSR4 and Gus control were determined. VSR4-C1A overexpression showed comparable TuMV CP and viral RNA level compared with GUS control, but much less than wild type VSR4 overexpression treatment. These data provide additional evidence supporting that VSR4-6K2 interaction is critical for TuMV infection.

**Part III – Minor Issues: Editorial and Data Presentation Modifications**
Reviewer #1: (No Response)

Reviewer #2: Chloroplast fluorescence is hardly visible in Fig 3C 48h. It is difficult to detect the signal also in 3E Col FM4-64 and avr4 mutant mCherry ARA Q69I panels.

**Responses:** We have prepared a new Fig 3C (upper channels), which zoomed in a region in previous Fig 3C (48 h) to show similar size of auto-fluorescent chloroplasts with that in lower panels (72 h). Fig.3E has been fixed and representative colocalizations of 6K2-GFP with enlarged LE labeled by FM4-64 or mCherry-ARA7^Q69L^ were indicated with white arrows.
